# Evaluation by Expert Dancers of a Robot That Performs Partnered Stepping via Haptic Interaction

**DOI:** 10.1371/journal.pone.0125179

**Published:** 2015-05-20

**Authors:** Tiffany L. Chen, Tapomayukh Bhattacharjee, J. Lucas McKay, Jacquelyn E. Borinski, Madeleine E. Hackney, Lena H. Ting, Charles C. Kemp

**Affiliations:** 1 Department of Biomedical Engineering, Georgia Institute of Technology, Atlanta, GA, USA; 2 Department of Biomedical Engineering, Emory University School of Medicine, Atlanta, GA, USA; 3 Department of Medicine, Emory University School of Medicine, Atlanta, GA, USA; 4 Department of Medicine, Atlanta VA Geriatric Research Education and Clinical Center, Atlanta, GA, USA; Politehnica University of Bucharest, ROMANIA

## Abstract

Our long-term goal is to enable a robot to engage in partner dance for use in rehabilitation therapy, assessment, diagnosis, and scientific investigations of two-person whole-body motor coordination. Partner dance has been shown to improve balance and gait in people with Parkinson's disease and in older adults, which motivates our work. During partner dance, dance couples rely heavily on haptic interaction to convey motor intent such as speed and direction. In this paper, we investigate the potential for a wheeled mobile robot with a human-like upper-body to perform partnered stepping with people based on the forces applied to its end effectors. Blindfolded expert dancers (N=10) performed a forward/backward walking step to a recorded drum beat while holding the robot's end effectors. We varied the admittance gain of the robot's mobile base controller and the stiffness of the robot's arms. The robot followed the participants with low lag (M=224, SD=194 ms) across all trials. High admittance gain and high arm stiffness conditions resulted in significantly improved performance with respect to subjective and objective measures. Biomechanical measures such as the human hand to human sternum distance, center-of-mass of leader to center-of-mass of follower (CoM-CoM) distance, and interaction forces correlated with the expert dancers' subjective ratings of their interactions with the robot, which were internally consistent (Cronbach's α=0.92). In response to a final questionnaire, 1/10 expert dancers strongly agreed, 5/10 agreed, and 1/10 disagreed with the statement "*The robot was a good follower*." 2/10 strongly agreed, 3/10 agreed, and 2/10 disagreed with the statement "*The robot was fun to dance with*." The remaining participants were neutral with respect to these two questions.

## Introduction

Partner dance is an effective rehabilitation intervention that relies heavily on haptic interaction between individuals. By haptic interaction, we mean any interaction through the sense of touch. Partner dance has been shown to improve balance, gait, functional mobility, and functional autonomy in people with Parkinson’s disease (PD) [1–3]. Participants undergoing partner dance therapy expressed enjoyment, satisfaction, improved well-being, and interest in continuing the therapy [2].

In partner dance, such as waltz, foxtrot, and tango, partners communicate haptically through constant physical contact in order to generate coordinated, whole-body motion. This contact is made via a configuration of their hands and arms called their frame. Effective communication is crucial to the interaction since partner dance is often improvised without a set sequence of steps [4], and evidence suggests that haptic information can be sufficient to perform partner dance with complicated movements [5, 6]. As such, partner dance may serve as a useful paradigm for scientific inquiry into physical human-human interaction [7].

Robots may be able to play beneficial roles in partner dance therapy, such as serving as dance partners, performing assessments of participants, and acting as scientific instruments with which to conduct research. Robots for upper and lower extremity rehabilitation have successfully performed comparable roles, from helping people recover function [8–10] to performing diagnostic assessments [11].

In this paper, we investigate the potential for a mobile manipulator with a wheeled base and compliant arms to perform a simple dance with a person. Specifically, in our study, the robot serves as the follower and the human as the leader in a partnered step during which the human walks backwards and forwards to a recorded drum beat while holding the robot’s end effectors.

The robot, Cody, is a general purpose robot that was not specifically designed for partnered dance. We built on our previous work in which we demonstrated that nurses could intuitively and effectively guide a robot by its end effectors [12]. For this paper, we use the same robot with a very similar admittance controller that commands the robot’s mobile base velocity to be proportional to the forces applied to the robot’s end effectors. By admittance controller, we mean any controller that commands a velocity based on a measured force.

A key distinction from previous research is that we conducted a formal study in which expert dancers haptically interacted with our robot and evaluated its performance. Much of the prior research with dancing robots has focused on visual interactions and participants who were not expert dancers [13–17]. This includes research on therapeutic robots that perform movements with or dance with older adults [18] and children [19] without physical contact. The more limited research that has looked at partner dance involving physical contact with humans has not involved formal evaluation with expert dancers [7, 20–23].

We focused on expert dancers for the following reasons: (1) effective rehabilitative partner dance has relied on expert dancers as instructors [1, 2], (2) expert dancers can perform the cooperative motor task with high skill due to years of training, which can serve as a model for future robotic performance, (3) our expert dancers have been instructors and hence are qualified to evaluate and communicate the quality of dance [24], and (4) expert dancers allow us to characterize skilled interaction prior to working with non-experts with balance and gait disorders, whom we expect to be more variable in their performance.

In addition to evaluating our robot’s performance using subjective measures, we identified objective biomechanical measures that correlate with favorable subjective dance experience. The biomechanical measures we identified can potentially help evaluate the performance of partner dance robots in the absence of expert dancers and quantify the effects of controller parameters on performance. We defined the partnered stepping task (PST) in order to establish a consistent, well-defined activity representative of dance for use in studies of whole-body physical coordination.

We also manipulated properties of the robot that might affect the haptic interaction during the PST to study their contributions to the interaction. In this work, we altered the robot’s arm stiffness and the admittance gain for the mobile base controller as part of a 2×2 within-subjects experiment. Varying properties of the robot resulted in varying levels of robot performance. This enabled us to correlate the participants’ subjective responses to objective biomechanical measures.

**Fig 1:**
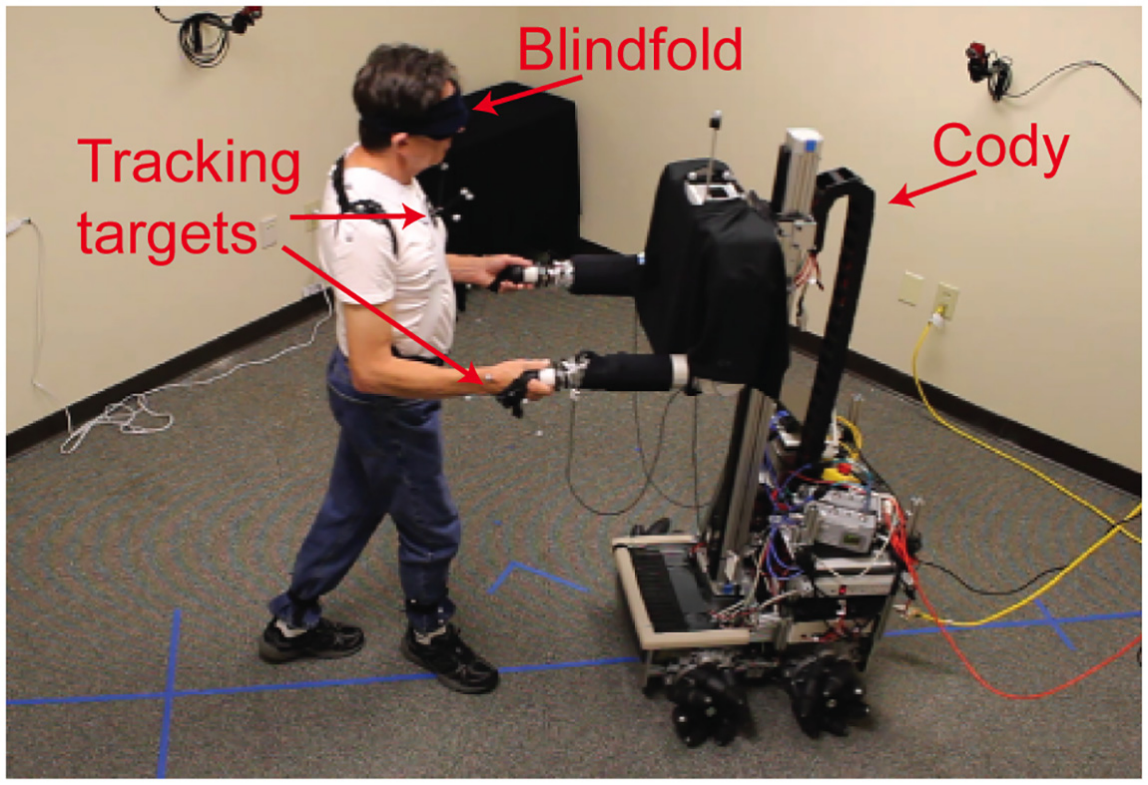
Experimental setup. An expert dancer leads the robot Cody during partnered stepping.

With this work, we make several contributions. First, we defined and conceived of the PST as well as created and validated a questionnaire to measure subjective dance quality. We believe that the information gained from these questions opens a needed window into the perceptions of experts within this particular human-robot interaction task, which has not been examined before in this manner. Second, we found that expert dancers were able to successfully perform the PST with a robot using only haptic interaction. This is a valuable result and contribution because though we hired the expert dancers based on their expertise, it was still possible for any of the participants to be unable to perform the task given the robot’s controller. Third, we found that a majority of expert dancers in our study interpreted the robot as following them well, and half found the robot to be fun to dance with. This is especially promising, given the straightforward controller for the robot’s mobile base and the simplicity of the PST. Fourth, we identified biomechanical measures correlated with subjective dance quality for the PST, including the human’s left hand to sternum distance, CoM-CoM distance, and forces at the hands. We did not measure the actual centers of mass. Instead, CoM refers to a point on the robot where one would expect the base of the neck to be located and the CoM for participants refers to the sternum motion-capture marker (See Fig 1). Fifth, we found that high admittance gain for the robot’s mobile base resulted in better subjective and objective dance performance. Taken as a whole, these contributions make progress toward designing an effective dance partner robot to provide rehabilitative therapy.

### Controller Design and Its Relationship to Other Human-Robot Dance Controllers

In this paper, we use a simplified version of a controller that we previously developed. The original controller enabled naive users to lead a robot through complex environments with high subjective and objective performance [12]. For our simplified version, we restricted the omni-directional robot to move forward and backward. The mobile base controller is a straightforward admittance controller that commands the velocity of the robot’s base to be proportional to the sum of the forces applied at the robot’s end effectors (see Section ‘Control of the Mobile Base’). In addition, for two of the four conditions we tested, we commanded the robot’s torque-controlled arms to have low proportional gains resulting in high compliance at the robot’s end effectors. Together, the complete system can be interpreted as behaving like a damper in series with a spring with the robot’s base motion emulating a damper and the robot’s arms acting as a spring.

As we discuss in detail below, our controller is similar to controllers used for other robotic dance partners. Two notable differences with respect to some other controllers are the lack of a simulated mass element and the simplicity of our controller. In the context of this study, both design choices have advantages. First, our system stops moving in the event that the human loses contact with the robot’s end effectors. This serves as a form of dead-man’s switch that reduces the chance of our ∼160kg robot colliding with or running over participants. This should be a consideration for naive users with impairments, which is our ultimate target population. Simulating a mass element would result in simulated inertia of the system and would require additional control elements to detect loss of contact and bring the robot to a halt. Second, while a number of research groups have developed elaborate controllers for human-robot dancing [20, 22, 23], careful evaluation of human-robot dance controllers through human studies has been limited. Our system is both simple and practical. The simplicity of our control system and the PST enabled us to focus our study on the relationship between biomechanical measures and subjective perceptions of dance. We varied key factors, such as the compliance of the robot’s arms, while avoiding potentially confounding factors, such as using a hybrid controller with discrete states. The practicality of our system increases the relevance of our results, since there would be fewer impediments to producing and deploying the technology in clinically relevant settings. The present study and our previous work [12] suggest that even this simple controller can result in both task success and positive interactions, which is promising for the future of robot-facilitated partner dance therapy.

In previous work, Gentry et al. used a force controller to allow a PHANToM haptic device to lead human participants’ hands through randomly sequenced trajectories [7]. A PD (Proportional-Derivative) controller fed a force back to the user’s hand through the device’s stylus with respect to a reference position and velocity.

Holldampf et al. used vector fields to enable a human-scale robot to lead a human through previously recorded trajectories from human dance couples [22]. They also scaled the robot’s trajectories based on forces measured at the hands to prevent the robot from dragging or pushing the human. The human interacts with the robot using an admittance-type haptic interface [25].

Takeda et al. developed a dance partner robot which could dance with a human by predicting the human’s next dance step and adapting the length of its own dance step stride based on the physical interaction [20].

Wang and Kosuge modeled the dynamics of a human and MS DanceR robot as a connected pair of inverted pendulums. They used the model to reduce the interaction force [23]. Their results suggested that the inverted pendulum model was better able to reduce the interaction force compared with a virtual force from the human’s estimated trajectory.

Bussy et al. [26, 27] enable the HRP-2 robot to carry a table with a human partner by using a trajectory-referenced admittance control law as well as a finite state machine to transition the robot’s motion primitives when both leading and following.

## Methods

This section focuses on the experimental methodology and the results of our experiments. For technical implementation details related to the robot and motion capture system, please refer to the appendix.

### Experimental Methodology

This section describes the recruitment procedure, experimental design, experimental procedure, and the objective and subjective measures.

#### Recruitment

We recruited 11 expert dancers via word of mouth, but only included data from 10 participants (N = 10) due to equipment failure in one experiment. Several participants were acquaintances of co-author Hackney. We obtained written informed consent from all participants according to our experimental protocol that was approved by the Institutional Review Boards of the Georgia Institute of Technology and Emory University. We required participants to meet the following inclusion/exclusion criteria: ≥ 18 years of age; ≥ 10 years of dance experience; ≥ 2 years of partner dance instruction experience; and no history of neurological disorders. We told participants that they would engage in partnered stepping and would interact with technology. We did not mention that the participants’ partner would be a robot so as to avoid biasing participants. The demographics of the participants were: 4 female, *M* = 43.5, *SD* = 12.4 years of age, *M* = 19.1, *SD* = 13.4 years dance experience, and *M* = 8.9, *SD* = 6.3 years partner dance instruction experience. The participants’ ages ranged from 26 to 66 years, years of dance experience ranged from 10 to 50 years, and years of partner dance instruction experience ranged from 2 to 25 years.

#### Experimental Design

We used a 2×2 within-subjects design with 3 repetitions of each treatment. We tested the independent variables of:

- *Gain*: high ($c=0.02\frac{m}{sN}$) vs. low ($c=0.01\frac{m}{sN}$)
- *Arm stiffness*: high ($2050\frac{N}{m}$) vs. low ($543\frac{N}{m}$)

We randomized the treatments in blocks of four and assigned three blocks to each participant for a total of (2 Gain) × (2 Arm stiffness) × (3 repetitions) = 12 trials per participant.

#### Procedure

The experiment took place at the Healthcare Robotics Lab (Atlanta, GA) from 6^th^ July, 2012 to 5^th^ September, 2012 in a 8.5 m × 3.7 m room. Initial paperwork and questionnaires took place just outside the room. An experimenter (first author of this paper) welcomed the participant and asked him or her to fill out a consent form, reimbursement paperwork, and a demographic information and pre-task questionnaire.

The experimenter told the participant that he or she would serve as the leader and that the robot would serve as the follower. The participant was instructed to interact with the robot performing a basic dance step, and to perform the step in a way that he or she was familiar with in partner dance. The experimenter instructed the participant on how to perform the task:

- Hold onto the robot’s end effectors.
- Lead the robot backward 3 steps, starting on the right foot.
- Collect the feet together by skimming the left heel above the floor and without shifting weight onto the left foot.
- Lead the robot forward 3 steps, starting on the left foot.
- Collect the feet together. (end of one cycle)
- Repeat until four cycles are completed.
- Hold pose at the end of the last cycle until the experimenter says that it is OK to let go of the robot.

We instructed participants to step at 42 beats per minute while listening to a synthesized drum beat at 84 beats per minute. We allowed them to take whatever size steps they were comfortable with while keeping inside the boundaries marked on the floor. The distance between these boundaries was 2.6 m.

The participant practiced the dance step without the robot until he or she was comfortable. The experimenters adjusted the robot’s height until the robot’s elbow height was comfortable for the participant. Then the participant practiced the dance step with the robot. During the practice session, we set the robot’s arm stiffness and gain settings corresponding with the first treatment in the randomization for that participant.

Once the participant was comfortable performing the task, the experimenter placed the motion capture reflective markers on the participant. The experimenter stated that the participant would perform the task with the robot 12 times and that the robot may or may not respond differently each time. The experimenter asked the participant to read a hard copy of the dance quality questionnaire along with a glossary (Tables 1 and 2) and told the participant that he or she would be given this questionnaire after each of the trials. The experimenter told the participant that his or her task was to focus on the interaction with the robot and to keep the questionnaire questions in mind.

**Table 1:** Glossary of dance terminology.

**Term**	**Definition**
Frame	A stable but flexible configuration of the arms and bodies of both the partners. In this experiment, the frame comprises the arms and bodies of you and the robot, connected at the hands.
Connection	Using the frame to transmit information through the hands in the form of direction, distance, and rotation, as well as intensity of movement, e.g., velocity and accent.
Timing	The coordination of movement to a pattern of beats that have a strong relationship with the phrasing, tempi, and beats of a musical selection.

This glossary is used for terms in the Dance Quality Questionnaire (Table 2).

Participants wore a blindfold and were asked to close their eyes while wearing the blindfold for all of the trials. After the completion of a trial, the participant removed the blindfold and completed the dance quality questionnaire on a computer. We provided a hard copy of the glossary (See Table 1) for reference next to the computer. We offered a snack and water to the participants during waiting times between trials.

After the participant completed the trials, the experimenter removed the tracking markers from the participant’s body and then asked the participant to complete a final questionnaire and a final interview. The entire experiment took approximately 2.5 hours.

#### Defining the Partnered Stepping Task (PST)

For this work, we defined the PST as:

1. partners are human-scale and in upright, standing postures, facing each other,
2. partners are constantly physically coupled through the frame (involving the upper limbs and torso),
3. haptics is the primary mode of interaction between the partners,
4. the whole bodies (e.g. centers-of-mass (CoMs)) of both partners move across the ground with humans performing overground walking,
5. one partner is designated as the “leader” who directs the motion of the partners,
6. at least one partner in the task maintains a cadence that is synchronized with an external auditory signal,
7. the leader moves his or her CoM in the forward/backward direction (1DoF) over a distance corresponding to multiple steps,
8. the other partner (e.g., the robot) is designated as the follower who interprets the cues provided by the leader.

Moving together while physically contacting one another is a key aspect of partner dance. The Partnered Stepping Task (PST) is a simple task representative of basic coordinated motions involved in partner dance. For example, Moore, a leading authority in ballroom dance technique, states that “To be able to walk properly in a forward and backward direction is the basis of ballroom dancing” [4].

In our experiment, items 1 and 2 of the definition of the PST would be satifised if the participants correctly executed the procedure in Section ‘Procedure’. Item 3 is satisfied due to the design of Cody’s mobile base controller where only forces are used as input as well as the fact that the participants are blindfolded and do not receive auditory feedback from the robot. Furthermore, item 5 of the PST is satisfied since the participants are instructed to serve as the leader and the robot is only capable of following by reacting to forces at the end effectors. Thus, if items 1, 2, 4, 6, 7, and 8 are satisfied during the execution of the interaction, the PST would be defined as successfully completed. We defined biomechanical measures that indicated satisfactory performance of several of these remaining items in Section ‘Biomechanical Measures of Dance’.

#### Subjective Measures of Dance

In order for the expert dancers to perform subjective evaluations of their haptic interactions with the robot, we needed appropriate instruments (e.g. a questionnaire). Although visually-based judging criteria of solo dance and partner dance exist from the perspective of a third party [24, 28–30], an instrument focusing on haptic evaluation does not exist to the best of the authors’ knowledge. In this work, based on consultations with experts and literature sources [31], we developed the dance quality questionnaire shown in Table 2.

**Table 2:** Dance Quality Questionnaire.

**Category**	**Question: The robot…**
Motor Intent	maintained connection well.
	was easy to communicate with.
	understood the direction in which I wanted it to go.
	understood the speed at which I wanted it to go.
	understood how far I wanted it to go.
Motor Performance	was easy to move with.
	maintained its frame well.
	responded with good timing to mine.
	was too heavy.
	moved in the direction in which I wanted it to go.
	moved at the speed at which I wanted it to go.
	moved how far I wanted it to go.
Motor Skill	did not rush me.
	gave me “just enough” space to move together well.

We asked participants to respond to these questions after each experimental trial. Responses are measured using 5-point scale where 1 = “Strongly Disagree,” 3 = “Neutral,” 5 = “Strongly Agree.” The questions can be considered to fall into the three categories noted on the left column of the table, although this information was not provided to the participants.

The questions are measured using 5-point Likert items where 1 = “Strongly Disagree,” 3 = “Neutral,” and 5 = “Strongly Agree.” We also used the accompanying glossary of terms shown in Table 1 for which we adapted some terms from [32]. We administered a final questionnaire at the end to assess the participant’s overall experience. The final questionnaire was composed of the following 5-point Likert items: (1) “The robot was fun to dance with.” (2) “I was dancing with the robot.” and (3) “The robot was a good follower.”

#### Biomechanical Measures of Dance

**Fig 2:**
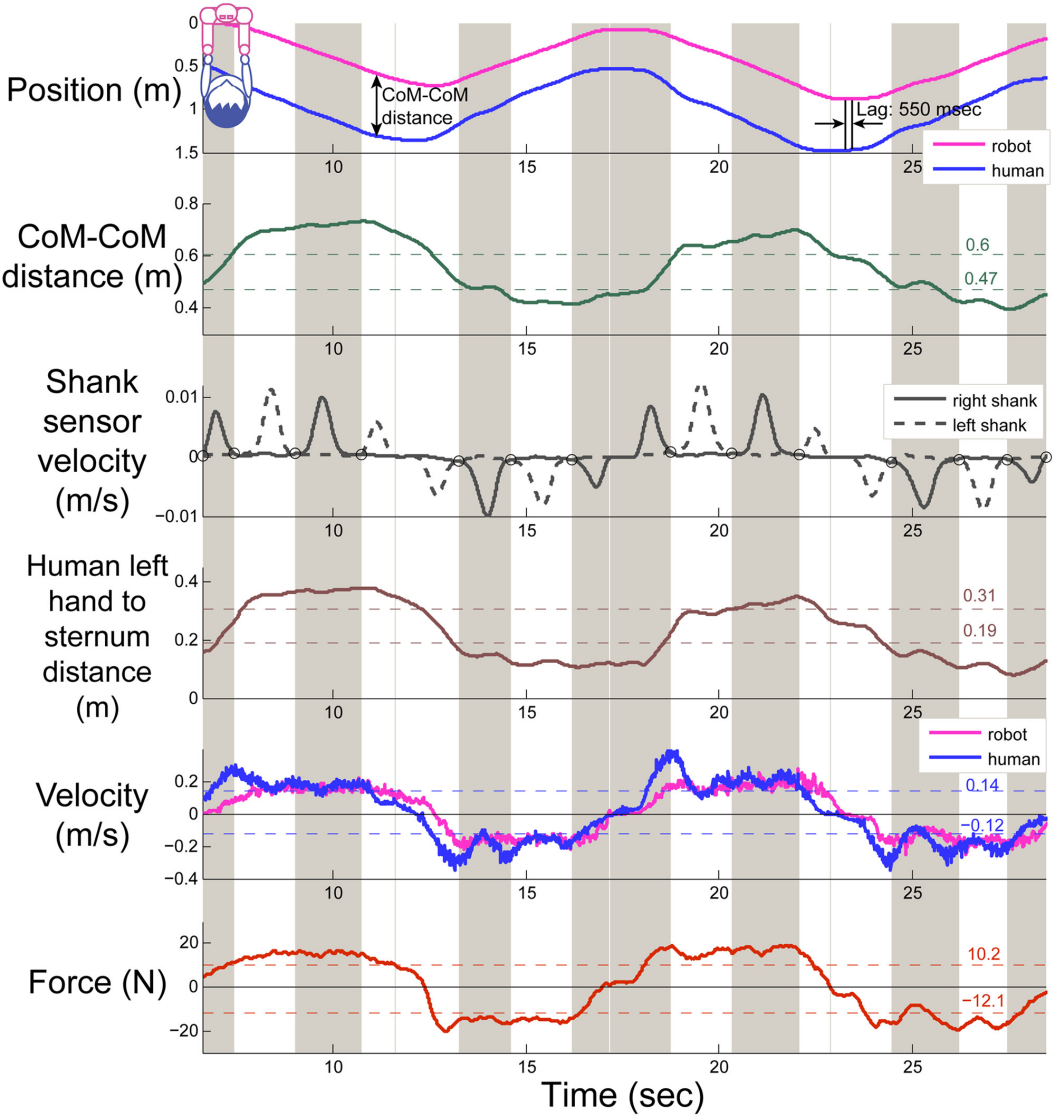
Biomechanics of human-robot partnered stepping. Example data from two cycles of one trial from one participant. Gray and white bars indicate intervals of time when right and left feet were on the ground, respectively. The experimental treatment for this trial was low gain, low stiffness.

We used kinematic and force data from each trial to objectively characterize measures of synchrony between the expert dancers and the robot, Cody, that would correspond with successful performance of the PST. We computed the mean force (N) at the hands, the velocity ($\frac{m}{s}$) of the human and robot partner, CoM-CoM distance (m), CoM-CoM variability (standard deviation of CoM-CoM distance) (m), human left hand to sternum distance (m), and human left hand to sternum distance variability (standard deviation of human hand to sternum distance) (m). We computed each measure separately during the phases of each trial in which the human partner was walking forward and in which the human partner was walking backward. We compute the lag time (lag) by cross correlating the robot’s position as a function of time and the human’s position as function of time, where position is a scalar. We determined the zero-crossings of the velocity of the right and left shank markers to determine when the participants placed their feet on the ground (shown as black circles in third plot of Fig 2). Using this information, we computed the average time between each of the participant’s footfalls to measure the participant’s average cadence (s) during each trial. We also computed the cadence variability (standard deviation) (s), cadence root mean square (RMS) (s), and cadence mean-squared error (MSE) (s) with respect to footfalls at 42 bpm corresponding with the instructions provided by the experimenter.

We expected that these measures would be correlated with responses to the dance quality questionnaire (Table 2). Several of these measures may correspond with the objective in partner dance to move together while allowing the leader to have enough space to be comfortable initiating direction and speed changes (i.e., low variability of human hand to sternum and CoM-CoM distance, low lag). The leader may also wish to do so using a minimal amount of force.

Furthermore, several of these measures have been used in previous studies on human-robot partner dance [20, 23] as well as in a study on the regulation of interpersonal distance between a pair of humans in a forward/backward walking task [33]. We expect that these baseline measures will enable future comparisons with participants who may have balance disorders (as done in [34–36]) or lower dance skill level.

#### Statistical Analyses

To determine the effects of the gain and robot arm stiffness factors, we performed a two-way, repeated measures ANOVA with 3 repetitions on the responses to the dance quality questionnaire as well as the biomechanical measures. We did not correct for multiple comparisons. We also performed one-sample *t*-tests on the responses to the final questionnaire, comparing them with a response level of 3.

**Fig 3:**
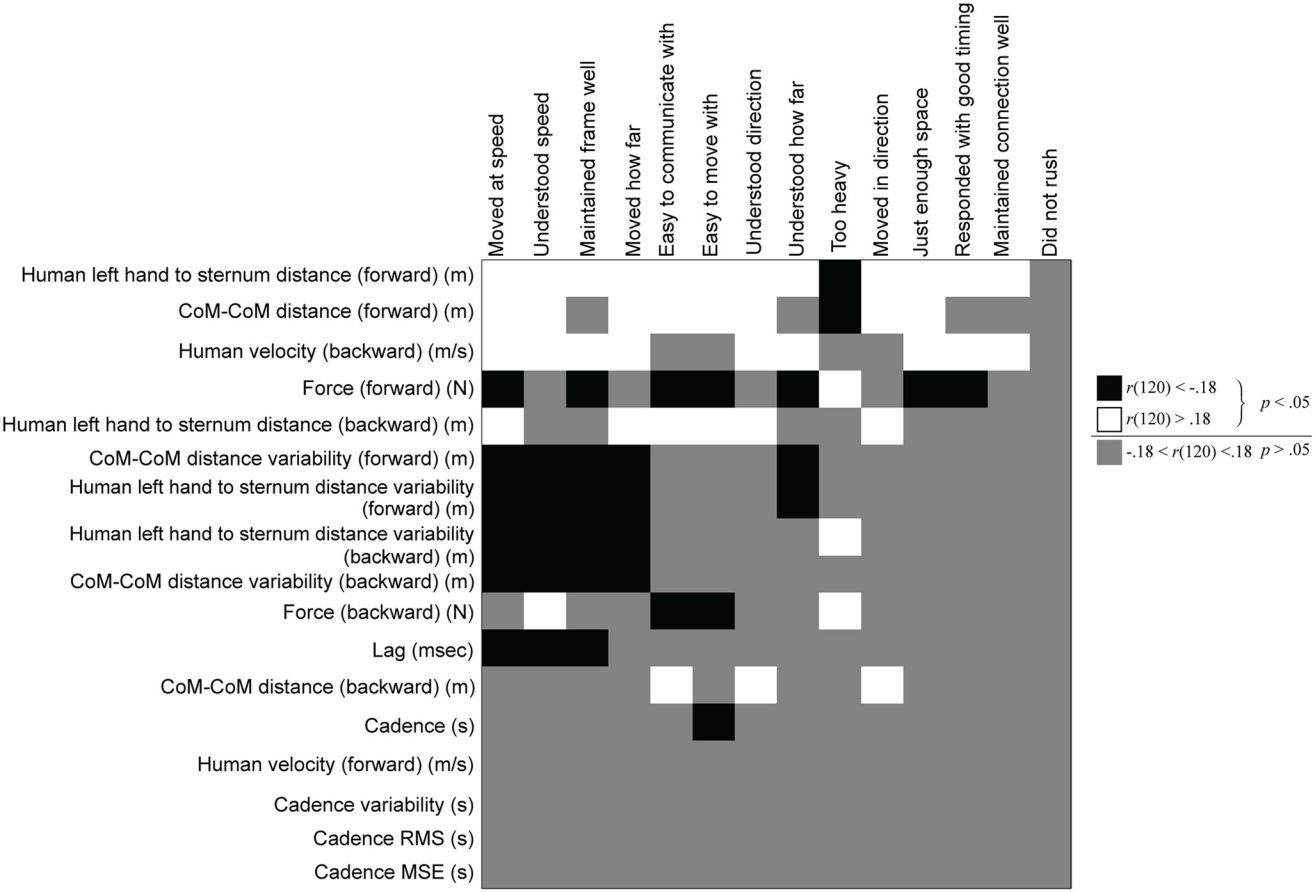
Correlation between subjective and objective measures. Biomechanical measures are listed on the rows and subjective measures are listed on the columns in descending order of number of significant correlations. Black and white boxes denote significant negative and positive Pearson’s correlations coefficients, respectively. Gray denotes non-significant correlations. For example, with increasing lag, participants report that the robot follower moves and understands the leader’s motor intention less well. Also, as the interaction force increases, the participants report that the robot follower becomes less easy to communicate or move with. Similarly, as the variability in hand-sternum / CoM-CoM distance increases, the follower understands or moves according to the leader’s motor intention less accurately. Interestingly, cadence variability, RMS, and MSE values have very little correlation with any of the subjective responses. Such useful insights are possible through this correlation matrix between the biomechanical measures and subjective responses.

We also calculated Pearson’s correlations between the biomechanical measures and responses to the dance quality questionnaire. Correlations that were significant at *α* = .05 corresponded to a Pearson’s correlation *r*(number of trials = 120)<-.18 for significant negative correlations and *r*(number of trials = 120)>.18 for significant positive correlations, denoted in Fig 3 as black and white colored squares, respectively.

We performed psychometric analyses on the responses to the dance quality questionnaire to assess its reliability and validity which are fundamental aspects of an accurate measurement instrument [37]. We computed a Cronbach’s alpha value to measure the internal consistency of the 14-item dance quality questionnaire. We also computed intra-class correlation coefficients (ICCs) to measure the inter-rater reliability among participants as well as the test-retest reliability among the three repetitions of the treatments. For these values, we referred to the general cutoff of .8 as indicating good consistency and reliability [38, 39] but considered lower values as acceptable due to the low number of participants.

## Results

We present the results with respect to the contributions of this work and include suggestions for improved interaction from the participants.

### Expert dancers successfully engaged in partnered stepping with a robot

Expert dancers interacted with the robot Cody according to the specifications of the PST in Section ‘Defining the Partnered Stepping Task (PST)’. In general, items 1 and 2 of the definition were satisfied by our observations that the participants completed the task procedure as described in Section ‘Procedure’, and items 4, 7, and 8 were satisfied by our observation of the biomechanics (example shown in Fig 2) that the CoMs of the human and robot moved in the forward/backward direction with multiple steps taken before each direction change. However, there were brief instances of apparently unstable interactions involving the robot oscillating back and forth. If the experimenter observed unstable motion, she would ask the participant to release the robot’s end effector or lighten his or her grip on the end effector. Otherwise, the trial would continue as usual. An inspection of the motion capture and force data found that 5 out of the total 120 trials appeared to exhibit brief instability. The only other experimenter intervention that we documented involved asking participants to stay within a marked region of the room, so that the motion capture system would be able to observe the interactions. This was also rare.

Furthermore, the average lag time of the robot behind the human was M = 224, SD = 194 ms across all conditions. This lag result was similar to results in [33] that reported average time lags ranging from 220 to 290 ms between human leader-follower pairs using visual cues. Also, the robot maintained a relatively consistent CoM-CoM distance as shown in the position trajectory in Fig 2 where CoM-CoM distance ranged from 0.39 to 0.72 m, M = 0.54, SD = 0.06 m across all conditions with relatively low variability (standard deviation is 6 cm). Item 6 was satisfied due to the participants averaging M = 1.41, SD = 0.02 s between each step across all conditions which is within one standard deviation from the expected 1.43 s per step cadence of the external auditory signal played at 42 bpm.

### Some expert dancers agreed that the interaction was fun and similar to dancing

**Fig 4:**
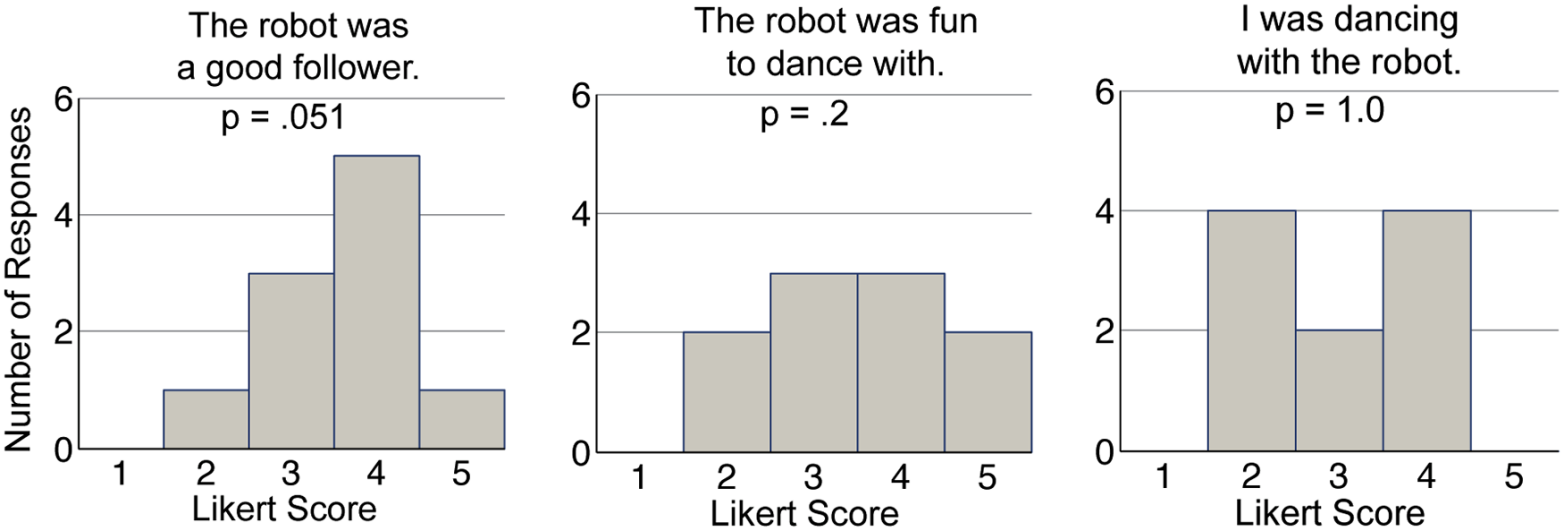
Final questionnaire responses regarding overall experience. Response level of 1 = “Strongly Disagree,” 3 = “Neutral,” 5 = “Strongly Agree.” *p*-values show results from one-sample *t*-tests comparing with a response level of 3.

As shown in Fig 4, 60% of the participants agreed (responded with a response level of 4 (“Agree”) or 5 (“Strongly Agree”)) that the robot was a good follower, 50% agreed that the robot was fun to dance with, and 40% agreed that the interaction with the robot was similar to dancing. While none of the distributions of responses were significantly greater than a response level of 3 (“Neutral”), these results are promising in that a majority felt that the robot was able to follow while at least a portion of the participants felt that the interaction was actually fun and similar to dance.

Participants generally agreed with the statement: “The robot was a good follower.” (*M* = 3.6, *SD* = 0.8, Fig 4).

- Of the 6 participants who responded with a response level of 4 (“Agree”) or 5 (“Strongly Agree”), (60%) mentioned that the robot had good connection, speed, and timing.
- Of the 3 participants who responded with a response level of 3 (“Neutral”) (30%), 1 participant expressed the robot was slow to react or was too heavy, and the participant had to “[work] hard.”
- Only one participant disagreed (response level of 2 (“Disagree”)) (10%). This participant stated that she had to make her frame more rigid or apply more force during some of the trials, and mentioned that the robot lagged or resisted too much to the direction or speed that she wanted.

Participants also responded favorably to the statement: “The robot was fun to dance with.” (*M* = 3.5, *SD* = 1.1).

- 5 of the participants (50%) responded with a response level of 4 (“Agree”) or 5 (“Strongly Agree”).
  3 of these participants compared the robot with a human in a positive way. For example, one participant stated: “I didn’t quite expect a robot to follow as well/better than some human dancers.” 2 participants mentioned that the experiment was “different” while 1 participant said it “engaged my curiosity.”
- 3 of the participants (30%) responded with a response level of 3 (“Neutral”). 1 of these participants mentioned that the task was simple and allowed him to think about the robot’s “reaction,” while another participant said that the task “was neither fun or not fun” since the direction of the task did not vary and felt that the robot was “mechanical.” The third participant stated that the interaction was “once in a lifetime” and “high-tech” but that the robot did not always follow smoothly.
- 2 of the participants (20%) disagreed (response level of 2 (“Disagree”)) and both mentioned that the interaction or robot was “novel.” 1 of these participants felt that the interaction was “monotonous” but that the “variety” of the interaction came from moving with the robot.

The responses to the statement “I was dancing with the robot” were split as shown in Fig 4 (*M* = 3.0, *SD* = 0.9). 8 of the 10 responses mentioned that “dancing” requires timing with the beat of the music and the partners moving together. The consensus was that the interaction constituted “limited” dancing.

- Of the 4 participants (40%) that responded with a response level of 4 (“Agree”) or 5 (“Strongly Agree”), 2 of the participants mentioned the robot responded well to signals given. 1 of the participants mentioned that dance requires emotion, implying that it was lacking. 2 of the participants described the movement as “technically dancing.”
- 2 of the participants (20%) responded with a response level of 3 (“Neutral”). 1 of these participants mentioned that since he was used to Argentine tango which is improvisational, the lack of variety in steps caused him to say that the interaction met the “minimum requirements”.
- 4 of the participants (40%) responded with a response level of 2 (“Disagree”). 1 of these participants mentioned the lack of variety of movement while another participant felt a lack of “creativity and freedom” during the interaction and was not “enjoyable.”

**Fig 5:**
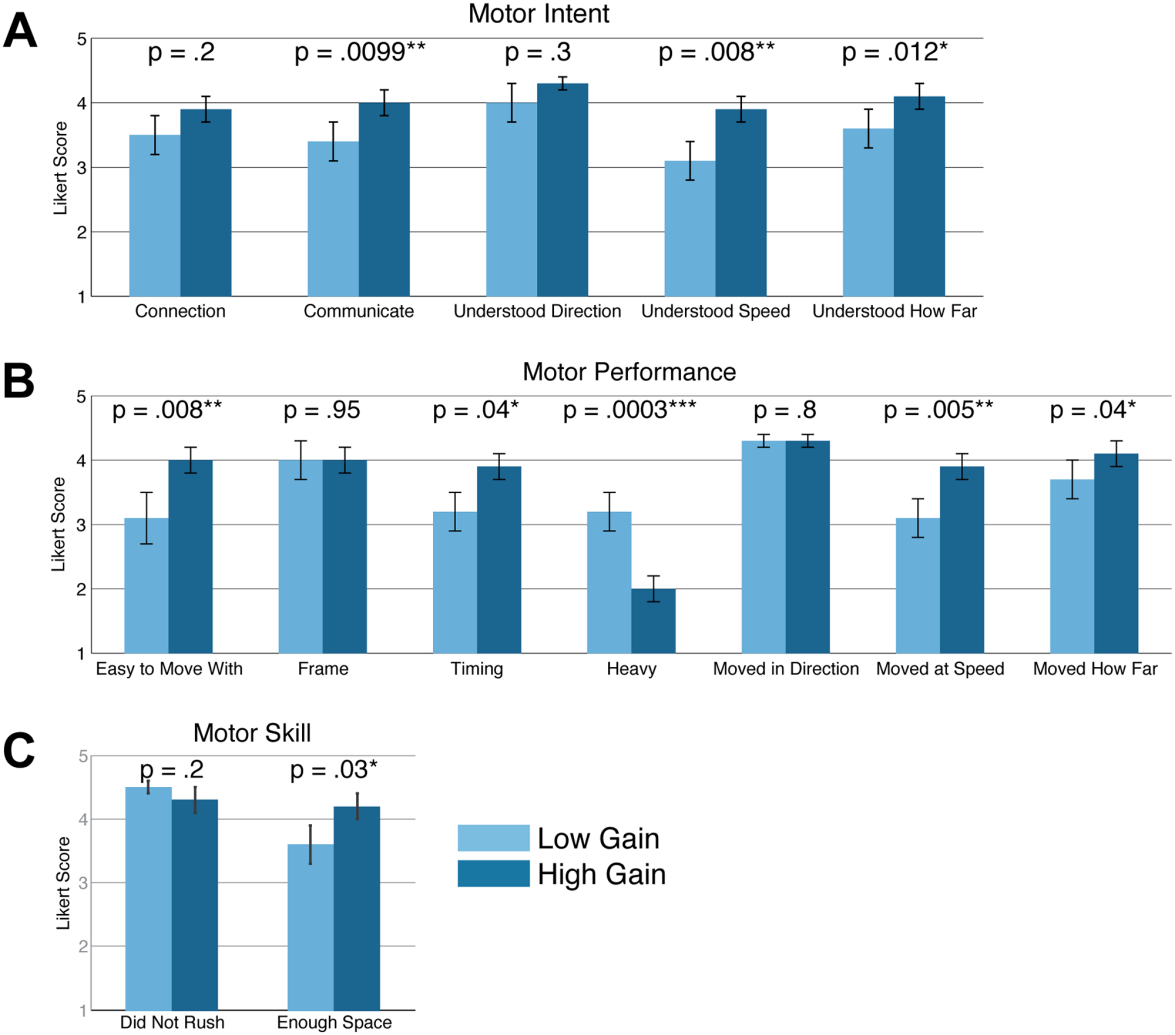
High admittance gain results in higher subjective dance performance. (A) Motor intent, (B) Motor performance, (C) Motor skill. Bars show mean and standard error. Response level of 1 = “Strongly Disagree,” 3 = “Neutral,” 5 = “Strongly Agree.”

The responses to the dance quality questionnaire (shown according to the gain factor in Fig 5) indicated that the expert dancers rated the robot’s subjective performance favorably on average. The response levels averaged above 3 (“Neutral”) for positively valenced questions and averaged below 3 (“Neutral”) for negatively valenced questions.

### Biomechanical measures correlate with subjective dance quality

The dance quality questionnaire had excellent internal consistency (Cronbach’s *α* = 0.92) across participants, treatments, and repetitions. Similarly, the test-retest reliability was good with an ICC of 0.80. The inter-rater reliability was acceptable (ICC = 0.58) given the questionnaire’s purpose of having consistency as well as providing differing insight among raters. These results indicate that the dance quality questionnaire can potentially serve as a reliable and valid instrument to measure dance performance.

Open-ended responses from the final questionnaire supported the validity of the dance quality questionnaire as developed from dancing literature and consultation with expert dancer and co-author Hackney. 7 of the 10 participants (70%) stated that the glossary of dance terminology used in the dance quality questionnaire was “concise” or “well described.” 2 other participants also agreed with the definitions, but mentioned that the definition of “connection” was more “complex” than the one used. 1 of these 2 people mentioned that emotional aspects of connection were omitted from the definition while the other mentioned that “tension” as well as frame are combined to “create a connection.”

Overall, the results of the correlation analysis between the subjective responses and biomechanical data (Fig 3) consistently indicated that longer human hand to human sternum distance, larger CoM-CoM distance, faster human walking speed, less force at the hands, lower variability of human hand to human sternum distance, lower variability of CoM-CoM distance, less lag, and faster cadence are associated with more favorable ratings of subjective dance performance. These results were in line with our expected outcomes. These correlations also support the validity of the biomechanical and subjective measures.

### High gain was rated more favorably by expert dancers

The subjective and objective measures of dance revealed significant differences according to the gain factor where high gain yielded better perceived performance versus low gain. We did not observe significant differences in the subjective measures due to the stiffness factor, however, high stiffness yielded significantly lower lag time of the robot behind the human. Also, neither the gain factor nor stiffness factor had a significant effect on any of the cadence measures.

#### Gain

High gain resulted in significantly more favorable dance quality questionnaire responses (*p* ≤ 0.05, see Fig 5). According to measures of motor intent (Fig 5A), the high gain setting allowed the robot to communicate significantly better than the low gain setting (*F*(1, 9) = 10.6, *p* = .01, $\eta_{p}^{2}$ = 0.54). Similarly, participants rated the robot better able to understand the speed at which they wanted the robot to go at the high gain setting compared with the low gain setting (*F*(1, 9) = 11.6, *p* = .008, $\eta_{p}^{2}$ = 0.56). Participants also felt that the robot was better able to understand the distance they wanted the robot to go when at the high gain setting (*F*(1, 9) = 9.8, *p* = .01, $\eta_{p}^{2}$ = 0.52).

According to measures of motor performance (Fig 5B), high gain also allowed the robot to be significantly easier to move with (*F*(1, 9) = 11.3, *p* = .008, $\eta_{p}^{2}$ = 0.56), to move with significantly better timing (*F*(1, 9) = 5.7, *p* = .04, $\eta_{p}^{2}$ = 0.39), to seem significantly less heavy (*F*(1, 9) = 31.8, *p*<.001, $\eta_{p}^{2}$ = 0.78), and to be significantly better able to move at the speed (*F*(1, 9) = 13.7, *p* = .005, $\eta_{p}^{2}$ = 0.6) and at the distance the human wanted (*F*(1, 9) = 5.5, *p* = .04, $\eta_{p}^{2}$ = 0.38). According to one measure of motor skill (Fig 5C), the high gain setting allowed the robot to be significantly better able to give the human enough space (*F*(1, 9) = 6.8, *p* = .03, $\eta_{p}^{2}$ = 0.43).

**Fig 6:**
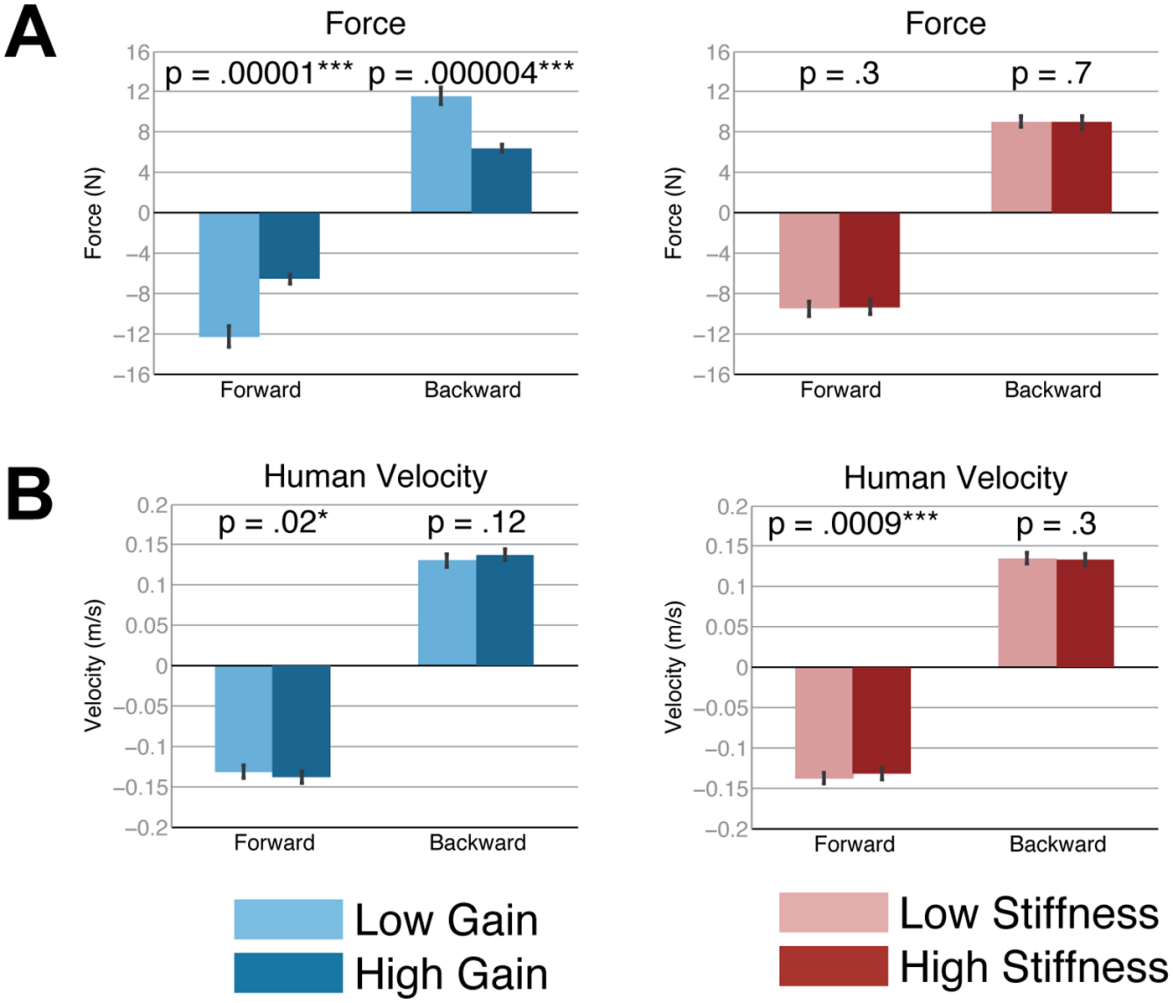
Humans adapt force input to maintain constant velocity. (A) Humans exert 0.53x and 0.56x force at the high gain setting compared to the low gain setting when walking forward and backward, respectively. (B) Humans maintain similar velocities across all conditions. Bars show mean and standard error.

The participants exerted significantly less force when interacting with the robot at the high gain setting versus the low gain setting (see Fig 6A), both when walking forward (*F*(1, 9) = 74.7, *p*<.001, $\eta_{p}^{2}$ = 0.89) and backward (*F*(1, 9) = 98.7, *p*<.001, $\eta_{p}^{2}$ = 0.92). Participants exerted 0.53x and 0.56x force at the high gain setting when walking forward and backward, respectively. These ratios are similar to the 2x ratio of the high admittance gain (0.02 m/sN) to the low admittance gain (0.01 m/sN) setting. Furthermore, since the participants moved at similar speeds across the gain conditions, it seems that the participants were adapting their force input to maintain a constant velocity according to the robot’s mobile base controller setting.

**Fig 7:**
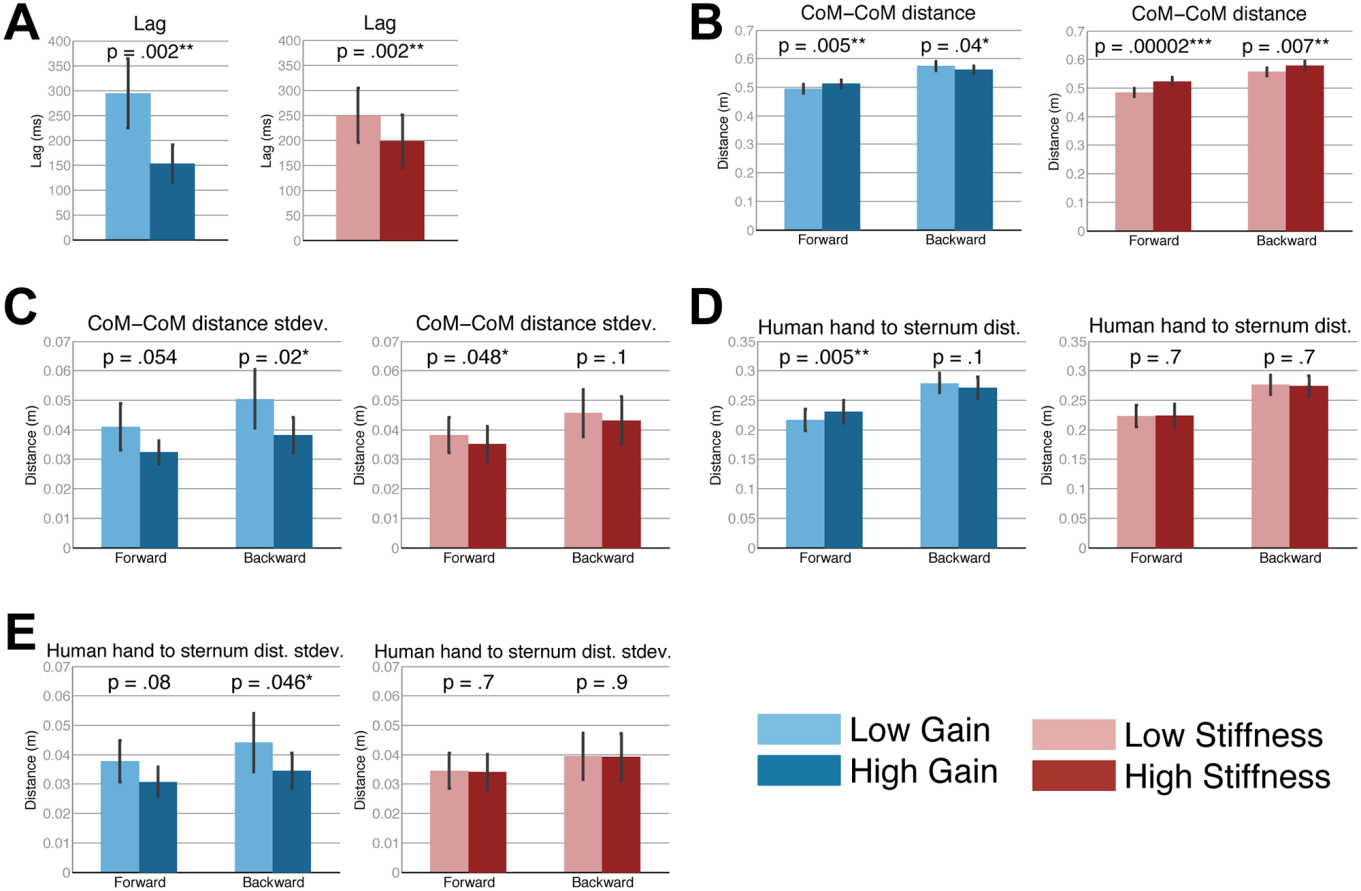
Biomechanical measures according to gain and stiffness. (A) Lag time of robot behind human, (B) CoM-CoM distance, (C) CoM-CoM distance standard deviation, (D) human left hand to sternum distance, (E) human left hand to sternum distance standard deviation. Bars show mean and standard error.

According to the objective measures, the robot lagged behind the human significantly less when at the high gain setting compared with the low gain setting (Fig 7A), (*F*(1, 9) = 17.7, *p* = .002, $\eta_{p}^{2}$ = 0.66).

The robot and human were significantly further apart from each other (larger CoM-CoM distance) when at the high gain setting when walking forward (*F*(1, 9) = 13.9, *p* = .005, $\eta_{p}^{2}$ = 0.61) and when at the low gain setting when walking backward (*F*(1, 9) = 5.5, *p* = .04, $\eta_{p}^{2}$ = 0.38). Similarly, the human hand to human sternum distance was significantly longer when at the high gain setting when walking forward (*F*(1, 9) = 13.3, *p* = .005, $\eta_{p}^{2}$ = 0.60). Thus, there may be a relationship between the human’s hand to human sternum and CoM-CoM distance. The participants maintained a more consistent CoM-CoM distance when at the high gain setting as indicated by the significantly lower variability (standard deviation) (*F*(1, 9) = 7.6, *p* = .02, $\eta_{p}^{2}$ = 0.46) for backward walking. This trend was echoed where the variability of the human’s left hand to sternum distance was significantly less variable at high gain for backward walking (*F*(1, 9) = 5.3, *p* = .046, $\eta_{p}^{2}$ = 0.37).

Experts rated higher gain as significantly more favorable according to several subjective measures of dance quality when performing the task. While this matched our expectations and common assumptions within the haptics community [40], we are unaware of previous work that has provided strong evidence for this in a controlled study in the context of dancing with a robot. Previous research has primarily focused on objective measures of tracking human motion with no clear relationship to subjective performance [7, 20–23]. While higher admittance gain (lower damping coefficient) resulted in better subjective performance, increasing this gain can result in system instability. Predicting whether a gain will result in a stable human-robot interaction is an open question, although researchers have made some progress in this area [41].

### Stiffness

None of the dependent measures from the dance quality questionnaire were significantly different due to the stiffness factor. However, according to the objective measures, the robot lagged significantly less behind the human when at the high stiffness setting compared with the low stiffness setting (Fig 7A), (*F*(1, 9) = 18.3, *p* = .002, $\eta_{p}^{2}$ = 0.67). Also, the CoM-CoM distance was significantly larger at the high stiffness setting both when walking forward (*F*(1, 9) = 65.6, *p* = <.001, $\eta_{p}^{2}$ = 0.88) and backward (*F*(1, 9) = 12.0, *p* = .007, $\eta_{p}^{2}$ = 0.57). Similarly, the variability of the CoM-CoM distance was significantly lower for high stiffness when walking forward (*F*(1, 9) = 5.2, *p* = .048, $\eta_{p}^{2}$ = 0.37).

### Expert dancers discuss benefits of the interaction and importance of other aspects of dance

Participants made a number of comments relevant to improving the robot. 4 participants suggested improving the robot’s performance regarding the “dampening,” improving mobility, connection, and responsiveness. 3 people suggested to improve the rhythm, for example, by varying the time signature of the music. 1 participant suggested varying the direction of motion. 1 person mentioned that being able to adjust the robot’s height made the interaction “[feel] good.” One participant suggested making the frame more like a “hug” configuration, while another suggested to replace the robot’s wheels with legs to “emulate humans more closely.”

Regarding their impression on being blindfolded during the experiment, 7 participants emphasized that it allowed them to “focus solely on the physical connection,” “sensation,” or “the feeling of the movement.” However, 1 person felt “awkward” while another felt that his “balance was compromised” but then said he wasn’t overwhelmed. 2 people stated that they were not accustomed to being blindfolded while dancing and that “visual perceptions” were missed. 1 of these 2 people, in addition to a third person, stated that blindfolds are used as a teaching tool in partner dance, but usually for the follower.

When asked whether the interaction felt different when walking backward vs. walking forward, participants were generally split or indifferent. 2 participants thought walking backward was easier while 3 participants thought it was easier to move forward. 2 of the 3 people who felt it was easier to walk forward felt that there was more pressure or resistance which made the connection better or that it helped stabilize the participant. 3 people felt there was no difference.

We asked participants: “What types of people might stand to benefit from a robot like this and why?” 3 people mentioned that the robot could be used as a diagnostic tool to “measure improvement,” for example, in strength and coordination. 1 person felt that it could be used to teach or train someone. Another participant felt that the robot might “make [exercise] more fun.”

## Discussion

The lack of significant differences among the measures according to the stiffness factor suggest that the difference in stiffness between the treatments might not be large enough to achieve any significant effect. Future investigation on more extreme levels of robot arm stiffness may yield significant results. The extent to which our results would generalize to other forms of partner dance, participants from other demographics, robots acting as the leader, and human-human partner dance remains an open question.

The results from this study demonstrated that the robot successfully followed expert dancers in partnered stepping according to subjective ratings by the participants as well as biomechanical measures indicating motion synchrony between the partners. We demonstrated that an admittance controller that is not specific to a particular dance enabled cooperative motion during partner dance using only haptic interaction.

We were also able to alter the expert dancers’ subjective dance experience with the robot by altering the robot’s admittance gain setting. A high admittance gain setting for the robot’s mobile base controller resulted in significantly higher subjective dance quality ratings as assessed by expert dancers. High admittance gain and high robot arm stiffness also improved the robot’s objective performance according to lag time behind the human leader. The role of arm stiffness in these interactions warrants further studies.

The correlation analysis between the subjective and biomechanical measures revealed that several biomechanical measures of synchrony could be used to objectively measure dance performance. Results indicated that the expert dancers rated their interaction more favorably with the robot when they were able to maintain longer and more consistent hand to sternum distance and inter-partner distance with the robot during the interaction. They also rated lower forces at the hands, lower lag time of the robot behind the human, and being able to step at a faster cadence as more favorable.

In this paper, we provide a framework for evaluating partnered stepping with a robot dance partner using only haptic interaction. By using a subjective dance quality questionnaire, we allowed the responses of expert dancers to guide the evaluation of our robotic system, the identification of biomechanical correlates of favorable performance, and the comparison of admittance gain and robot arm stiffness properties as it relates to dance performance.

This work has contributed toward developing a robotic platform that can intuitively engage in rehabilitative human-robot partner dance with end users. By continuing to investigate this human-robot partnered stepping paradigm, we can develop an understanding of the role of haptic interaction during two-person, whole-body motor cooperation tasks.

Please note that though one of our long-term objectives is for robots like this to serve as rehabilitation robots, for this study, we focused on our short-term objective of having a robot competently perform a simple dance step with able-bodied people. This is an important milestone towards a robot capable of safely and effectively interacting with people with impairments, such as people with Parkinson’s disease.

## Appendix

### Robot Implementation

This section describes the controllers used for this work. Our control system consists of a mobile base controller, which approximates a damper, as explained in Section ‘Control of the Mobile Base’ and robot arm controllers, which approximate springs, as described in Section ‘Control of the Arms’. We also describe the robot’s arm stiffness settings for the experiment as well as details of the motion capture system.

#### Robot Description

The robot Cody comprises: two 7 degree-of-freedom (DoF) arms from MEKA Robotics (MEKA A1), an omnidirectional base (Segway RMP 50 Omni), and a 1 DoF linear actuator (Festo) to allow vertical motion of the robot’s torso. The arms are anthropomorphic with series elastic actuators (SEAs) at each of the joints, which enable low-stiffness actuation. The robot’s wrists are equipped with 6-axis force/torque sensors (ATI Mini45). The robot is statically stable, weighs roughly 160kg, and must actuate its wheels in order to move.

#### Control of the Mobile Base

We used an admittance controller to control the motion of the robot’s mobile base in response to forces applied to the robot’s end effectors. The velocity commanded to the robot’s base in the forward/backward direction $\dot{x}$ was computed using the equation

$$\begin{array}{ccc} \dot{x} & = & c\cdot f_{tot} \end{array}$$ (1)

where *c* is an admittance gain (using the terminology of [42]) and *f*_*tot*_ is the sum of the forces at the robot’s end effectors in the forward/backward direction. The relationship between the force and velocity can be interpreted as a damper. The maximum commanded velocity was limited to 0.7 m/s in either direction, where 0.6 m/s is the minimum walking speed required for independent living [43]. We used a Kalman filter on force sensor readings below 3N. We also averaged the three most recent commands for $\dot{x}$ in order to reduce noise and smooth velocity transitions. The *gain* setting was an independent variable in the experiment for this paper where the admittance gain was set to $c=0.01\frac{m}{sN}$ and $c=0.02\frac{m}{sN}$ for the low and high gain settings, respectively (Section ‘Experimental Methodology’). The high gain setting was similar to the setting used in previous work with nurses [12].

We used a digital voice recorder to characterize the time delay between the onset of an applied force at the end effector and the onset of motion of the robot’s base. The average time delay for 5 trials for each of the treatment conditions were: *low gain, low stiffness*: *M* = 171, *SD* = 34 ms, *low gain, high stiffness*: *M* = 157, *SD* = 24 ms, *high gain, low stiffness*: *M* = 125, *SD* = 28 ms, and *high gain, high stiffness*: *M* = 109, *SD* = 6 ms.

#### Control of the Arms

**Fig 8:**
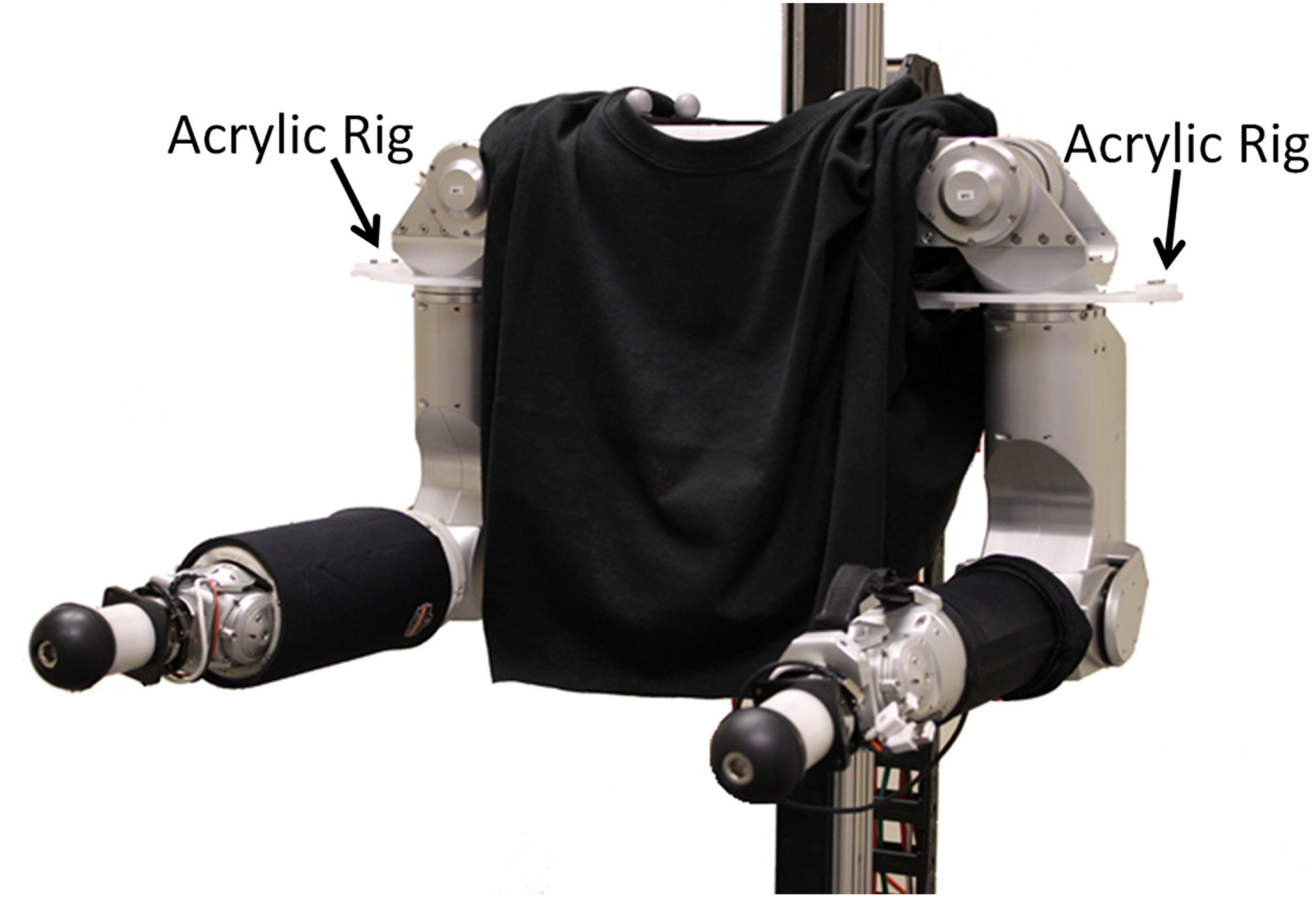
Acrylic rig used in the high stiffness condition. Black cloth sleeves were draped over the robot’s upper arms to conceal the presence or absence of the acrylic rig during the high stiffness and low stiffness conditions, respectively.

We commanded the torque-controlled arms to maintain a bent elbow posture throughout the interaction (see Fig 8). We set the stiffness for the shoulder and elbow joints in order to attain a desired stiffness at the end effector in the forward/backward direction. This stiffness primarily resulted from the elbow stiffness and one of the shoulder joints. We set the pitch and yaw wrist joints to be in a high-stiffness position control mode for all four conditions to keep the end effectors parallel to the forearms.

At the high stiffness setting for the independent variable of *stiffness*, we attached a custom-fit, laser cut acrylic rig (see Fig 8) to Cody’s upper arm in order to make the arms stiff in the forward/backward direction. In addition, we commanded high stiffness values at the shoulder and elbow joints. For the low stiffness setting, we removed the rig to allow the arm to rotate freely at the shoulder joint. In addition, we commanded low stiffness values at the shoulder and elbow joints. We placed a black t-shirt over the shoulders of the robot to cover the rig and thereby hide its presence or absence for the trials.

We measured the stiffness at the end effector for each of the conditions by displacing the end effector in the forward/backward direction while the robot was in the arm configuration used for the experiment. We measured the resultant forces at the end effector using the force/torque sensors and the displacements using the motion capture system. We took the slope of the line that best fit the force vs. displacement scatter plot under both stiffness conditions. We averaged the stiffness values from the left and right arms for each of the conditions obtaining a high stiffness of $2050\frac{N}{m}$ (*R* ^2^ = 0.91) and low stiffness of $543\frac{N}{m}$ (*R* ^2^ = 0.94).

#### System Characterization

**Fig 9:**
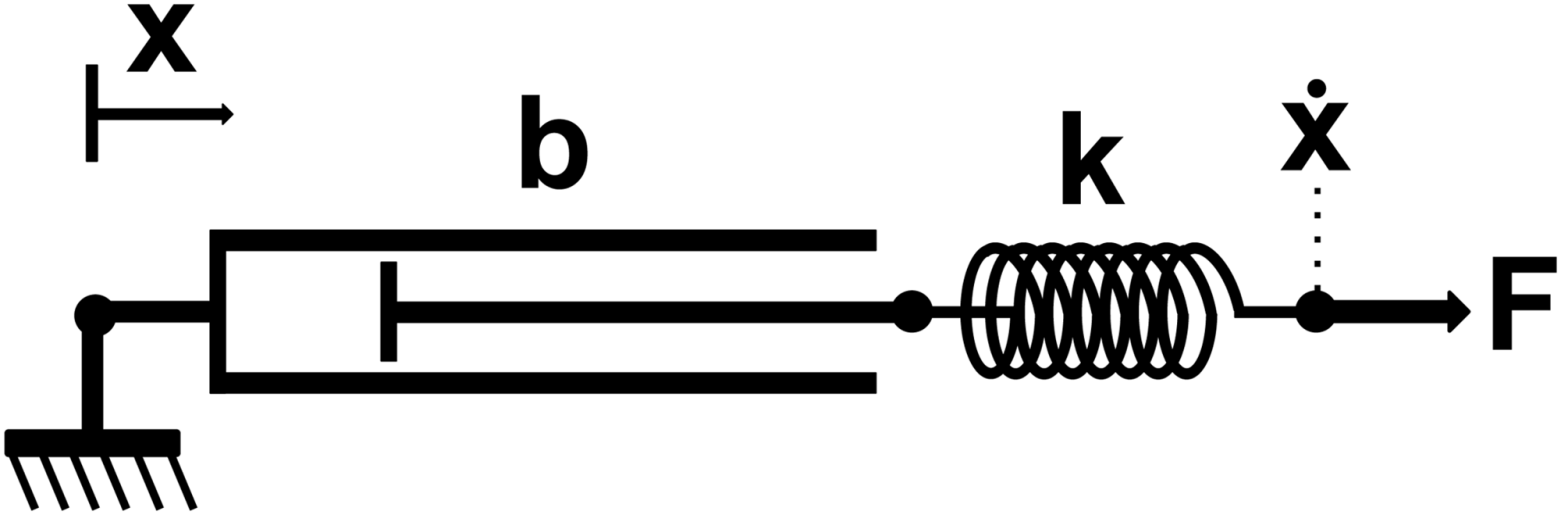
Model of the robotic system. The damper with damping coefficient b corresponds with the mobile base and the spring with spring constant *k* corresponds with the robot’s arm. *F* and $\dot{x}$ are the force and velocity at the robot’s end effector.

We created an idealized lumped-parameter model of the robot consisting of a spring in series with a damper, as shown in Fig 9. The spring models the robot’s compliant arms and the damper models the robot’s mobile base, which has a velocity that depends on the measured force. For this model, we set the spring constant $k=543\frac{N}{m}$ based on empirical measurements described above. We set the damping coefficient $b=100\frac{sN}{m}$, which is the reciprocal of the admittance gain of the mobile base controller that we programmed.

**Fig 10:**
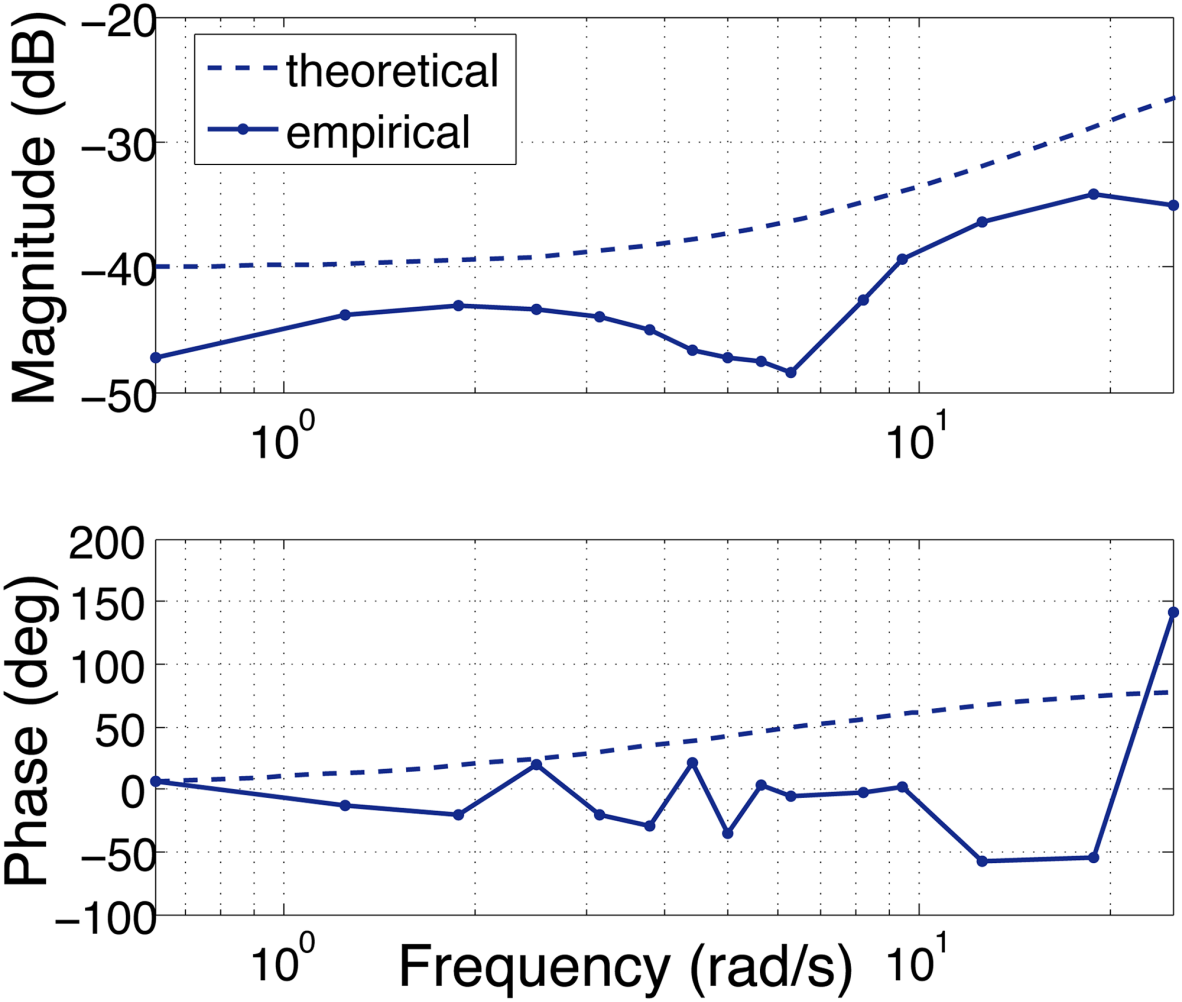
Bode plot for Low Gain, Low Stiffness. Input and output are force and velocity at the end effector, respectively. Empirical curve shows measured response of the robot. Theoretical curve shows the response of the ideal spring-damper model.

We also experimentally measured the system gain and phase for the low gain, low stiffness treatment by applying sinusoidal velocity inputs at the robot’s left end effector using a linear actuator at varying frequencies. We measured the force at the robot’s end effector as the system input and the velocity at the end effector as the system output. The results of the analysis are shown in the Bode plot in Fig 10.

The theoretical magnitude and phase plots of the transfer function of the spring-damper model are shown in Fig 10 using dashed lines. The damper dominates at low frequencies and the spring dominates at high frequencies. The empirical magnitude and phase plots are similar to the magnitude and phase associated with our idealized model of the system across all frequency ranges.

### Motion Capture

We tracked the motion of the human expert dancer and robot using the NaturalPoint OptiTrack motion capture system and Tracking Tools software (Corvallis, OR). We tracked the position and orientation of iotracker (Vienna, Austria) rigid body targets. Each is composed of four retro-reflective markers mounted on a base. We placed a rigid body target on the human’s sternum, shoulders, hands, and shanks using elastic straps (Fig 1). The individual shown in Fig 1 of this manuscript has given written informed consent (as outlined in PLOS consent form) to publish these case details. We placed one custom made rigid body target on the robot’s torso.
